# ClusterCAD 2.0: an updated computational platform for chimeric type I polyketide synthase and nonribosomal peptide synthetase design

**DOI:** 10.1093/nar/gkac1075

**Published:** 2022-11-23

**Authors:** Xavier B Tao, Sarah LaFrance, Yifei Xing, Alberto A Nava, Hector Garcia Martin, Jay D Keasling, Tyler W H Backman

**Affiliations:** Department of Chemistry, University of California, Berkeley, CA 94720, USA; Biological Systems and Engineering Division, Lawrence Berkeley National Laboratory, Berkeley, CA 94720, USA; Biological Systems and Engineering Division, Lawrence Berkeley National Laboratory, Berkeley, CA 94720, USA; Biofuels and Bioproducts Division, Joint BioEnergy Institute, 5885 Hollis Street, Emeryville, CA 94608, USA; QB3 Institute, University of California, Berkeley, CA 94720, USA; Department of Electrical Engineering and Computer Sciences, University of California, Berkeley, CA 94720, USA; Biological Systems and Engineering Division, Lawrence Berkeley National Laboratory, Berkeley, CA 94720, USA; Department of Chemical and Biomolecular Engineering, University of California, Berkeley, CA 94720, USA; Biological Systems and Engineering Division, Lawrence Berkeley National Laboratory, Berkeley, CA 94720, USA; Biofuels and Bioproducts Division, Joint BioEnergy Institute, 5885 Hollis Street, Emeryville, CA 94608, USA; Department of Energy Agile BioFoundry, Emeryville, CA 94608, USA; Biological Systems and Engineering Division, Lawrence Berkeley National Laboratory, Berkeley, CA 94720, USA; Biofuels and Bioproducts Division, Joint BioEnergy Institute, 5885 Hollis Street, Emeryville, CA 94608, USA; Department of Chemical and Biomolecular Engineering, University of California, Berkeley, CA 94720, USA; Department of Bioengineering, University of California, Berkeley, CA 94720, USA; QB3 Institute, University of California, Berkeley, CA 94720, USA; Novo Nordisk Foundation Center for Biosustainability, Technical University of Denmark 2800Copenhagen, Denmark; Center for Synthetic Biochemistry, Institute for Synthetic Biology, Shenzhen Institutes for Advanced Technologies, Shenzhen, China; Biological Systems and Engineering Division, Lawrence Berkeley National Laboratory, Berkeley, CA 94720, USA; Biofuels and Bioproducts Division, Joint BioEnergy Institute, 5885 Hollis Street, Emeryville, CA 94608, USA

## Abstract

Megasynthase enzymes such as type I modular polyketide synthases (PKSs) and nonribosomal peptide synthetases (NRPSs) play a central role in microbial chemical warfare because they can evolve rapidly by shuffling parts (catalytic domains) to produce novel chemicals. If we can understand the design rules to reshuffle these parts, PKSs and NRPSs will provide a systematic and modular way to synthesize millions of molecules including pharmaceuticals, biomaterials, and biofuels. However, PKS and NRPS engineering remains difficult due to a limited understanding of the determinants of PKS and NRPS fold and function. We developed ClusterCAD to streamline and simplify the process of designing and testing engineered PKS variants. Here, we present the highly improved ClusterCAD 2.0 release, available at https://clustercad.jbei.org. ClusterCAD 2.0 boasts support for PKS-NRPS hybrid and NRPS clusters in addition to PKS clusters; a vastly enlarged database of curated PKS, PKS-NRPS hybrid, and NRPS clusters; a diverse set of chemical ‘starters’ and loading modules; the new Domain Architecture Cluster Search Tool; and an offline Jupyter Notebook workspace, among other improvements. Together these features massively expand the chemical space that can be accessed by enzymes engineered with ClusterCAD.

## Graphical Abstract

**Graphical Abstract:**
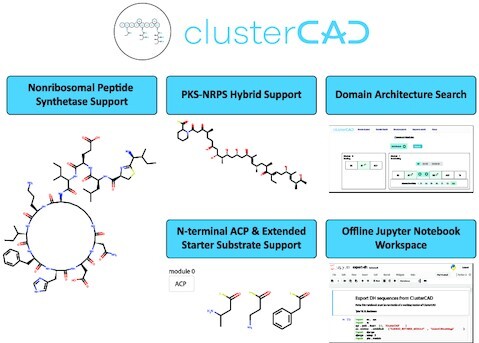
New features in ClusterCAD 2.0 include support for Non-Ribosomal Peptide Synthetases (NRPSs), PKS-NRPS hybrids, a new Domain Architecture search tool, expanded PKS starters, and a Jupyter notebook system for programmatic access.

## INTRODUCTION

Polyketide and nonribosomal peptide natural product derivatives are ubiquitous throughout clinical settings as antibiotics, antifungal, and immunosuppressants, among other applications. Initially inspired by the goal of developing novel drug analogs, polyketide synthase (PKS) and nonribosomal peptide synthetase (NRPS) engineering has since become a major focus within synthetic biology and has expanded to include the biological production of industrially-relevant commodity chemicals as well as small molecule unnatural products (1–11).

With some exceptions, the sequence of domains and modules in Type I modular PKSs and NRPSs deterministically predict the structure of the produced polyketide or peptide. Both PKSs and NRPSs can include multiple polypeptide subunits, which in turn are constituted by modules. PKS modules are composed of at minimum a set of ketosynthase (KS), acyltransferase (AT), and acyl carrier protein (ACP) domains, but can also alter the functional groups present on the nascent polyketide using optional ketoreductase (KR), dehydratase (DH), enoyl reductase (ER), and methylation (MT) domains. KS domains catalyze a decarboxylative Claisen condensation of an AT-selected dicarboxylic acid extender with an acyl starter or intermediate. Reduction of the β-ketone may also occur in the presence of KR, DH, and ER domains to form a β-alcohol, $\alpha$,β-unsaturation, or a fully saturated β-methylene, respectively. Polyketides are then typically released from a PKS via a thioesterase (TE) domain, which catalyzes the hydrolysis of the ACP-polyketide thioester into a free carboxylic acid or cyclic lactone, or by a reductase (R) domain which releases the product as an aldehyde or alcohol. The polyketide may then undergo further tailoring reactions to form the final PKS product.

NRPSs are very similarly organized, with modules at minimum composed of a set of condensation (C), adenylation (A), and peptidyl carrier protein (PCP) domains. C domains catalyze an amide condensation of an A-selected amino acid extender with an acyl starter or intermediate (12). Condensed peptides can be further modified utilizing optional epimerization (E), formylation (F), and *N*-methyltransferase (nMT) domains among others in order to change amino acid stereochemistry from L to D, add a *N*-formyl group to the N-terminal amino acid (13), or add a *N*-methyl group to the amide, respectively. Peptides are then also typically released from a NRPS via TE domains, catalyzing the hydrolysis of the PCP-peptide thioester into a free carboxylic acid, cyclic lactone, or cyclic lactam. The peptide may then undergo further tailoring reactions to form the final NRPS product.

The deterministic, collinear, and modular nature of both PKS and NRPS clusters present valuable opportunities for combinatorial megasynthase engineering. In these clusters, the sequential organization of domains within modules and clusters directly determine the arrangement and composition of chemical intermediates produced. Furthermore, the modularity of megasynthase clusters render great flexibility for module rearrangements during engineering, generating a vast chemical space of feasible products utilizing combinatorial PKS and NRPS systems. Notable examples within this immense chemical space include petroleum-derived hydrocarbons, fatty acids, small molecule polyketide drugs, small molecule peptide/polypeptide drugs, some terpenes/terpenoids, and their derivatives or analogs.

**Figure 1.**
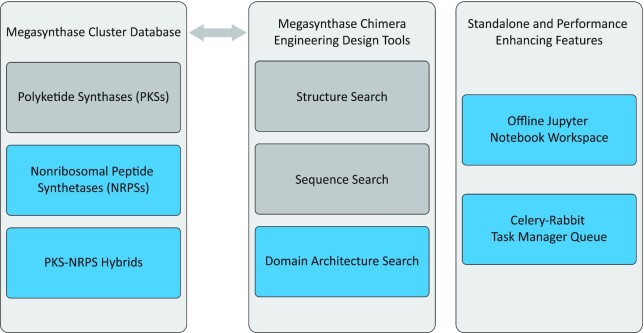
Overview of ClusterCAD 2.0’s suite of features. Features new to ClusterCAD 2.0 (NRPSs, PKS-NRPS Hybrids, Domain Architecture Search, Offline Jupyter Notebook Workspace, and Celery Task Manager Queue) have been indicated in blue, while pre-existing features have been indicated in gray.

**Figure 2.**
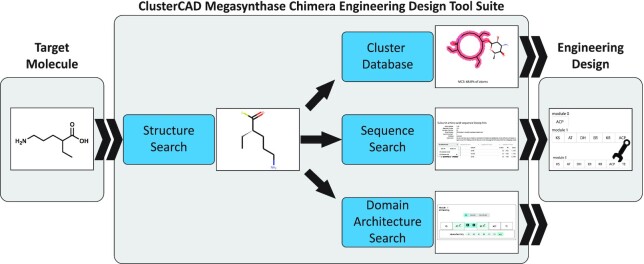
Sample PKS/NRPS megasynthase chimera engineering design workflow using ClusterCAD 2.0. First, a truncated PKS starting point is identified based on a desired target molecule using the structure search tool. Donor reductive/modification loops or domains are then identified by inspection of the cluster database, utilizing the sequence search tool and/or via the Domain Architecture Search Tool to inform modifications to the truncated PKS rendering it capable of generating the target molecule. The same tools can later be utilized to locate reductive/modification loops for domain swaps to optimize activity for increased target compound production.

Many tools have been developed to assist with general retrobiosynthesis and metabolic engineering (14–18), but to our knowledge none have been developed to specifically assist PKS and/or NRPS engineering. ClusterCAD, however, informs PKS and NRPS engineering by providing a paradigm, database, and tools in which PKS and NRPS parts can be identified for combinatorial biosynthesis based on amino acid sequence similarity or target-intermediate substructure similarity tools, maximizing the chance of a functional engineered chimeric PKS or NRPS (19). ClusterCAD’s paradigm is also strongly bolstered by experimental evidence demonstrating strong correlation between product formation and atom-pair chemosimilarity (as determined by ClusterCAD’s structure search tool) between donor and recipient module substrates in PKS reductive loop swaps (20). ClusterCAD has also been used for providing structural insight into DH domain substrate selection via sequence analysis (21) and predicting AT substrates (22), among other applications. In this regard, ClusterCAD provides a flexible toolkit to inform the design and engineering of chimeric megasynthases (Figures 1 and 2).

## NEW FEATURES AND METHODS

### NRPS support

**Figure 3.**
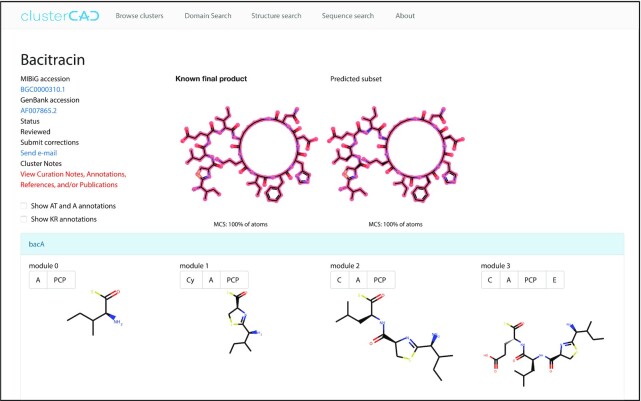
ClusterCAD entry for bacitracin, a newly added NRPS cluster. The ClusterCAD page for each cluster now contains new interface features including the Reviewed/Unreviewed Cluster indicator suggesting manual correction status, cluster notes providing relevant publications, and an option to toggle domain annotations for both AT and A domains.

Previously in ClusterCAD, the only type of megasynthase available for chimeric design and engineering were PKS clusters consisting of KS, AT, KR, DH, ER, ACP, and TE domains. With ClusterCAD 2.0, NRPS clusters received support. This includes previously truncated PKS-NRPS hybrid clusters in addition to full NRPS clusters composed of condensation (C), adenylation (A), peptidyl carrier protein (PCP), heterocyclization (Cy), epimerization (E), formylation (F), N-methyltransferase (nMT), monooxygenase-interrupted adenylation (AOX), terminal reductase (R), and oxygenase-recruiting X (X) domains (Figure 3). Each domain simulates chemical reactions computationally using reaction SMARTS (https://www.daylight.com/dayhtml/doc/theory/theory.smarts.html) and the cluster loading pipeline was adapted to recognize these domains from annotations generated by antibiotics and Secondary Metabolite Analysis (antiSMASH) and configure them accordingly (23).

A domains, especially following Cy domains, are frequently found to contain an oxidation or reduction domain looped out of itself used to catalyze the oxidation or reduction of thiazoline or oxazoline moieties into thiazols and oxazols or thiazolidines and oxazolidines, respectively. This catalytic activity was reflected by establishing A domain types as ‘oxidation’, ‘reduction,’ or ‘none’ if it performs neither additional reaction.

Similarly, C domain types vary depending on the nature of the reaction they catalyze, and were annotated through the antiSMASH output. Some C domains that begin chain extension at the N-terminus were designated as the ‘Starter’ type, C domains catalyzing the condensation between L amino acids as the ‘LCL’ type, C domains catalyzing the condensation between a D and L amino acid as ‘DCL’, C domains performing condensations within a glycopeptide NRP cluster as ‘Glycopeptide’, and dual condensation/epimerization C domains both performing a condensation followed with epimerization of the amino acid as ‘Dual.’

Through the addition of NRPS domains, 16 previously truncated or newly added PKS-NRPS hybrid clusters such as rapamycin (24), FK520 (25), and meridamycin (26) became fully supported.

### Database expansion and updates

ClusterCAD 2.0 provides users with a more expanded, comprehensive, and informative database. ClusterCAD 2.0’s database contains a total of 531 PKS, PKS-NRPS hybrid, and NRPS clusters, 183 of which have been manually curated based on experimental evidence from published literature—a ∼2.5-fold increase from ClusterCAD’s original 72 manually curated clusters.

The database expansion also features improvements to assess the quality of selected PKS and NRPS components for PKS–NRPS engineering experiments. A new Reviewed/Unreviewed Cluster Indicator designates whether a cluster has been manually corrected with experimental evidence or has not yet been manually reviewed and is displaying default antiSMASH annotations. When browsing the list of clusters, users have the option to either browse reviewed clusters only or both reviewed and unreviewed clusters. Similarly, when utilizing ClusterCAD’s substructure search or sequence search tool, users may select whether they prefer to search either reviewed clusters only or both reviewed and unreviewed clusters. By including unreviewed clusters, but marking them as such, we enable users to access a much larger set of potential megasynthase parts for engineering than those that have been well studied and characterized.

Furthermore, Cluster Notes now showcase any curation notes, annotations, references, or publications relevant to the cluster. Every cluster newly added in ClusterCAD 2.0 provides relevant publication(s) used for curating and annotating each cluster in addition to any curation notes for the user when considering the cluster or its components for engineering efforts. Cluster Notes are displayed at the top left of each cluster, and if available, will open a pop-up window with the aforementioned information.

### Improved database dependencies and pipeline

ClusterCAD 2.0’s database utilizes cluster annotations generated using antiSMASH 6.0 and cluster information using MIBiG (Minimum Information about a Biosynthetic Gene Cluster) 2.0 (27). Updated multiple times since the original ClusterCAD’s usage of antiSMASH 3.0, antiSMASH 6.0 features improved NRPS adenylation domain substrate recognition, efficiency improvements, and module detection that power ClusterCAD 2.0’s improved ability to generate clusters based on the organization of detected modules or prediction of NRPS domain substrates (23,28).

ClusterCAD 2.0 also boasts a powerful new pipeline tailored for generating clusters from antiSMASH 6.0’s annotations. In addition to support for updated dependencies, the new pipeline also accommodates the importation of more clusters. To reflect the diversity of PKS and NRPS clusters in nature (29), the new pipeline supports a multitude of new loading module configurations, including N-terminal ACPs as a standalone module, CoA ligase (CAL) based CAL-ACP, CAL-PCP, AT-ACP, and A-PCP domain organizations. Furthermore, the new pipeline also automates structure-informed adjustments to PKS reductive domain activities to reliably reflect the correct biosynthesis steps. Incomplete module annotations or natural cluster evolution will infrequently cause modules to contain reductive domains lacking other reductive domains critical for its prerequisite reaction. For example, the first module in the AbsB2 subunit of the Abyssomicin cluster contains a DH domain (as predicted by antiSMASH) but lacks the KR domain necessary to generate the subsequently dehydrated alcohol. In this case, the new pipeline recognizes the lack of a domain required for a prerequisite reaction and inactivates any subsequent reductive domains. This prevents reductive domains lacking a suitable precursor substrate from being annotated as active.

These dependency updates and pipeline improvements provide users with higher quality and more numerous clusters (over 7× as many including unreviewed clusters).

### Expanded database structure diversity via newly supported cluster and domain features

Several additional features pertinent to new substrate loading domains and chain-release mechanisms were added to ClusterCAD 2.0. Standalone N-terminal acyl carrier protein (ACP) domains are a structural feature frequently observed in many PKS and NRPS clusters, where the N-terminal ACP domain loads a structural intermediate generated from a series of preceding non-modular reactions. N-terminal ACP loading substrates vary by complexity and functional group composition between clusters, providing an extraordinary level of chemical diversity nonexistent with traditional PKS-NRPS starter or extender substrates.

Additionally, polyketide chain-releasing thioesterase (TE) domains now support lactam ring formation. In most PKSs, TE domains found at the C-terminal end of polyketide assembly lines release the polyketide by catalyzing the formation of a free carboxylic acid via thioester hydrolysis or a lactone via cyclization. However, in some PKS clusters like cremimycin (30) and many NRPSs like bacitracin (31), the TE domain catalyzes the formation of a lactam via cyclization. This new functionality has rendered support for a multitude of clusters boasting cyclic lactams and cyclic peptides, both of which are chemical moieties frequently found in both natural products and industrially useful synthetic chemicals. These new chemical motifs and clusters featuring them provide many novel substrates and domains, presenting as attractive targets for PKS or NRPS engineering.

Terminal reductase (R) domains are also newly supported. In an NADPH-dependent reaction, R domains can reduce the R-polyketide or R-peptide thioester linkage into an aldehyde or alcohol depending on the domain's target level of reduction. In clusters like Spumigin E and Coelimycin, peptide or polyketide chain release depends on R domain mediated reductive release, therefore providing additional clusters and unique functional groups that can be taken advantage of in PKS-NRPS engineering.

Support for N-terminal ACP domains, TE domain lactam formation, and R domain terminal reductive release immensely enhances the chemical diversity of new structure possibilities within ClusterCAD 2.0. This expanded cluster and chemical library can be easily harnessed for PKS-NRPS engineering using the chemical structure search tool linked to ClusterCAD 2.0’s vastly expanded database or simply by browsing clusters.

### Domain Architecture Cluster Search tool

Due to the deterministic nature of PKSs and NRPSs, finding specific domain arrangements within natural clusters is immensely useful as they provide valuable starting points and structural insight for PKS and NRPS engineering. In this regard, ClusterCAD 2.0 offers the Domain Architecture Cluster Search (e.g. Domain Search), a new tool enabling the querying of specific customized arrangements of PKS domains (NRPS support is under development). The tool allows users to assemble sequences of domains, specify domain attributes (e.g. AT substrates and KR stereochemistry), and submit the query to search the ClusterCAD database for matches. Matching or similar cluster hits are then displayed by similarity score for review. Reviewing cluster hits can yield insight into the ordering, sequences, and nascent chemical chain structures of natural clusters within the ClusterCAD database resembling the query cluster domain arrangement and attributes. Furthermore, the tool can be used to elucidate structural alterations (e.g. substrate domain or reductive loop swaps) necessary for engineering existing natural clusters into producing a modified target chemical structure. This tool works by converting the sequence of domains in a megasynthase enzyme into a sequence of Unicode text characters, each representing a unique chemical configuration of a domain (e.g. a ketoreductase or KR of Type A1). We then compare the query to enzymes in the database by computing the Levenshtein distance (32). The Levenshtein distance is a uniquely useful similarity metric for megasynthase engineering because it reports ‘distance’ as a real world number of domain changes (e.g. delete, replace, or add) required to convert one enzyme design into another, and provides a list of these modifications suitable for guiding the automated or manual design of chimeric enzymes. The Domain Architecture Cluster Search therefore serves as an invaluable tool for both discovering natural cluster starting points and providing structural insight for PKS and NRPS engineering.

### Offline Jupyter Notebook Workspace

ClusterCAD 2.0 also offers an offline Jupyter Notebook workspace environment for software development useful for prototyping both standalone and ClusterCAD database-dependent programs. Support for an offline Jupyter Notebook workspace allows users to generate and run custom scripts and programs within ClusterCAD, extending the utility of ClusterCAD’s database beyond currently available tools and vastly increasing ClusterCAD’s potential for assisting PKS and NRPS engineering or other research applications. For instance, users might find it useful to extract libraries from the database with more complex rules beyond those possessed by preexisting ClusterCAD tools, especially if they are concerned with particular domains or domains possessing specific domain attributes (e.g. KR type). In this regard, the user could compose and run a script exporting sequences fulfilling specified conditions into a FASTA file. The offline Jupyter Notebook workspace therefore substantially increases database versatility by supporting a multitude of applications both within and beyond chimeric megasynthase engineering, ranging from using alignment dissimilarity predictions to inform domain swap experiments to generating megasynthase phylogenetic trees. Additionally, users can utilize the offline Jupyter Notebook workspace to manage and run any standalone Python scripts, Jupyter Notebooks, and programs.

### Celery task manager

To improve the efficiency of running tool queries, ClusterCAD 2.0 features a Celery distributed task queue. The Celery task queue enables long running tool queries to run in the background, without timing out the users web connection. This provides the potential for much more complex queries, and lays a foundation for long running compute intensive analysis tools which we plan to add in the future.

## CONCLUSION

To the best of our knowledge, ClusterCAD remains the only comprehensive web-based toolkit developed specifically to aid in chimeric megasynthase design. ClusterCAD 2.0’s improvements reinforce this mission through vast increases in cluster database size, expanded megasynthase diversity, additional powerful search tools, and informative interface features.

## DATA AVAILABILITY

ClusterCAD 2.0 can be freely accessed at: https://clustercad.jbei.org/. The ClusterCAD source code is available at https://github.com/JBEI/clusterCAD, released under a BSD style license. The source code also allowed users to run their own local/offline instance of ClusterCAD, including the integrated Jupyter Notebook Workspace.
